# A Case of Cutaneous Blastic Plasmacytoid Dendritic Cell Neoplasm on the Chest

**DOI:** 10.7759/cureus.70288

**Published:** 2024-09-26

**Authors:** Rana Tokioka, Natsuko Saito-Sasaki, Yu Sawada

**Affiliations:** 1 Dermatology, University of Occupational and Environmental Health, Kitakyushu, JPN

**Keywords:** blastic plasmacytoid dendritic cell neoplasm, case report, dermoscopy, skin eruption, tumor

## Abstract

Blastic plasmacytoid dendritic cell neoplasm (BPDCN) is a rare and aggressive blood cancer that often presents with skin lesions and can involve other organs, including the bone marrow. Despite initial responses to treatment, most patients eventually experience disease progression. We report the case of an 82-year-old male with a red chest nodule, later diagnosed with BPDCN and acute myeloid leukemia (AML). Dermoscopy revealed reddish-purple dots, and a biopsy confirmed BPDCN. The patient responded to venetoclax and azacitidine but relapsed five months later. This case highlights the importance of early diagnosis of BPDCN and the utility of dermoscopy in this tumor, which can contribute to timely treatment and improved patient outcomes.

## Introduction

Blastic plasmacytoid dendritic cell neoplasm (BPDCN) is a rare and aggressive hematologic malignancy that originates from precursor plasmacytoid dendritic cells [1]. BPDCN is characterized by its unique clinical and pathological features, which most commonly present with skin lesions, including nodules, plaques, or tumors [2-4], often accompanied by systemic organ involvement, including bone marrow infiltration [5]. Despite its initial responsiveness to multi-agent chemotherapy, the majority of patients eventually develop resistance [6], leading to disease progression. This poor outcome underscores the urgent need for improved diagnostic and therapeutic strategies.

## Case presentation

**Figure 1:**
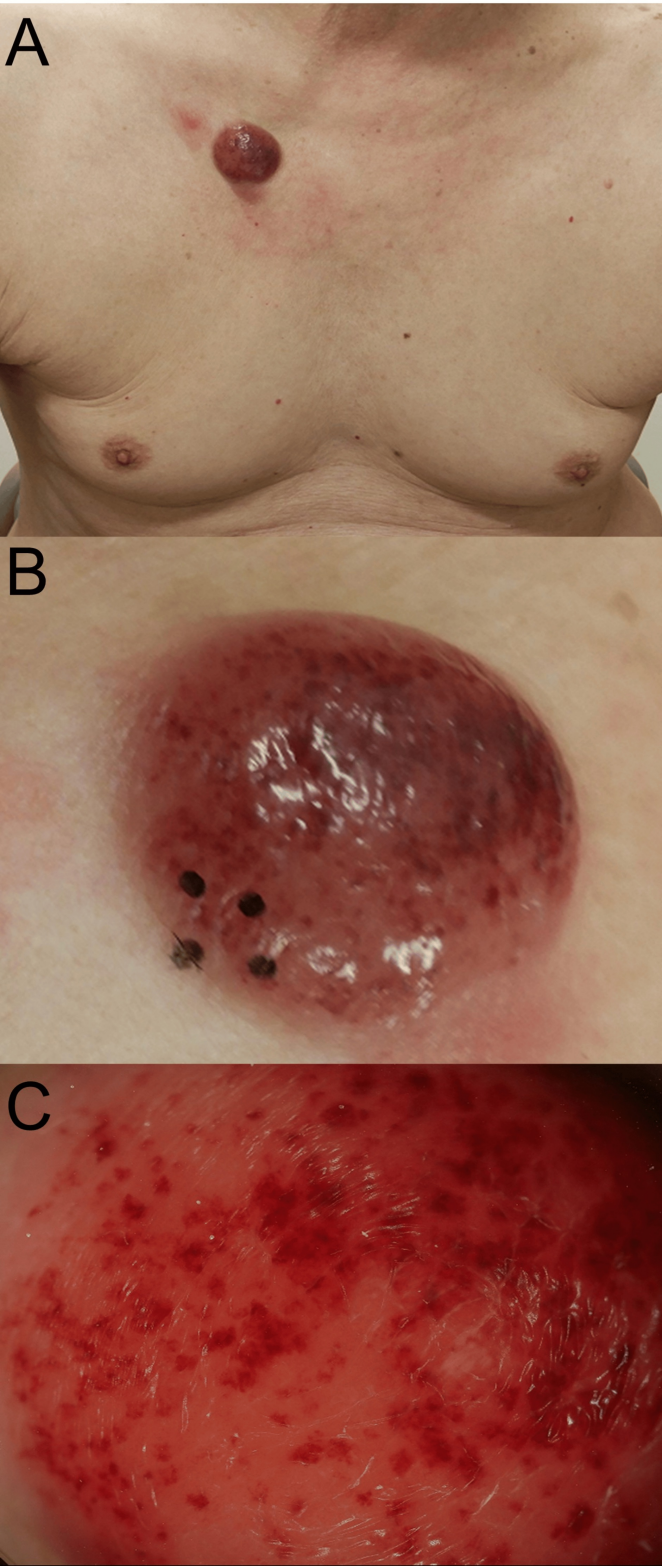
Clinical manifestation and dermoscopy findings. A) Clinical manifestation: a red dome-shaped tumor with a shiny surface observed on the right anterior thorax, 3 cm in diameter. (B) High Magnification View: Detailed observation of the tumor surface and its surrounding area. (C) Dermoscopy: Densely clustered reddish-purple dots, some merging together against a vivid red backdrop.

An 82-year-old male showed a red nodule that appeared on the right anterior chest two months prior to the first visit in our department; he was referred to our department for further evaluation and treatment. A red dome-shaped tumor with a shiny surface was observed on the right anterior thorax, 3 cm in diameter (Figures 1A-1B). There was no palpable axillary lymphadenopathy.

**Figure 2:**
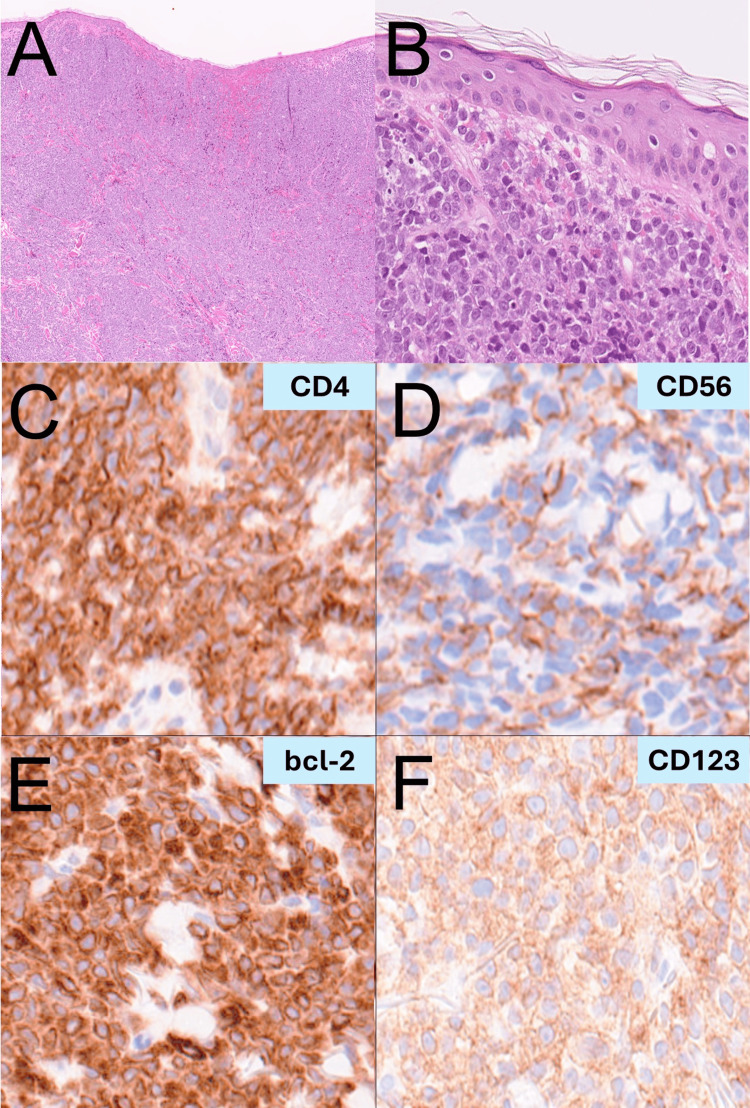
Histological examinations. (A) Histopathological examination at low magnification (×5): shows dense infiltration of atypical cells in the dermis and subcutaneous tissue with a clear Grenz zone separating the epidermis (H&E stain). (B) High magnification (×50): reveals blast-like atypical cells with irregular nuclei, fine chromatin, multiple nucleoli, and sparse cytoplasm proliferating in the dermis and subcutaneous tissue (H&E stain). (C-F) Immunohistochemical staining: tumor cells showing positivity for CD4 (C, ×100), CD56 (D, ×100), bcl-2 (E, ×100), and CD123 (F, ×100).

Dermoscopy revealed densely clustered reddish-purple dots, some merging together against a vivid red backdrop (Figure 1C). A skin biopsy taken from the tumor showed dense infiltration of atypical cells forming a sheet-like pattern in the dermis and subcutaneous tissue, with a clear boundary between the epidermis and underlying tissue, namely the Grenz zone (Figure 2A). High magnification revealed blast-like atypical cells with irregular nuclei, fine chromatin, multiple nucleoli, and sparse cytoplasm, proliferating diffusely in the dermis and subcutaneous tissue (Figure 2B). Extravasation of red blood cells was observed in the upper dermis (Figure 2C). Additionally, focal areas of small vessel proliferation and minor hemorrhages were observed within the dermis. The infiltrating atypical cells were positive for CD4, CD56, bcl-2, and CD123 (Figures 2C-2F), while negative for CD3, CD20, CD38, CD79a, and MPO.

Contrast-enhanced CT and positron emission tomography (PET)-computed tomography (CT) images revealed a 2.4 cm raised skin mass on the right anterior chest. No enlargement of supraclavicular, internal thoracic, axillary, pulmonary, or mediastinal lymph nodes was observed. PET-CT also showed increased uptake at the site of the mass, but no uptake in the lymph nodes. A hematology consultation was conducted, and a bone marrow examination revealed a slight increase in bone marrow blasts.

Based on the above findings, the patient was diagnosed with BPDCN; however, only 11.5% blasts were identified in the bone marrow aspiration. After diagnosis, the patient was admitted to the hematology department and treated with venetoclax plus azacitidine. The tumor rapidly shrank, and remission was achieved on day 21, allowing the patient to be discharged. However, five months after diagnosis, blasts were detected in the peripheral blood, and chemotherapy was resumed after reevaluation.

## Discussion

The skin manifestations of BPDCN typically present as purplish lesions, which can serve as a clue to diagnosis. With the advancement of dermoscopy techniques [7], these clinical manifestations of BPDCN may represent crucial findings during dermoscopy examinations. This report presents a case of BPDCN diagnosed from a skin tumor on the anterior chest of an elderly male patient, where densely clustered reddish-purple dots were identified via dermoscopy, corresponding to purplish lesions, on histological examination.

BPDCN is a hematologic malignancy originating from precursor plasmacytoid dendritic cells, classified under acute myeloid leukemia and related precursor neoplasms [1]. Given the poor prognosis and the rarity of the tumor [1, 8], early diagnosis is crucial for initiating evaluation and treatment. A previous study based on the clinical manifestations of the tumor revealed that purplish nodules were present in 73% of cases, while purplish bruise-like macules appeared in 12% [9]. In our case, histological analysis also revealed hemorrhages. Furthermore, dermoscopic examination reflected these findings, revealing densely clustered reddish-purple dots, some merging together against a vivid red backdrop. These dermoscopic findings might be helpful in identifying the tumor and could serve as clues for diagnosis.

As the differential diagnosis, we first considered cutaneous T-cell lymphoma (CTCL), especially mycosis fungoides, as a potential diagnosis, as it can present with reddish-purple lesions similar to those seen in BPDCN. However, the absence of characteristic dermoscopic patterns such as arborizing vessels or dotted vessels led us to exclude CTCL [10]. Histologically, CTCL typically shows epidermotropism [11], which was absent in our case. Instead, we observed a clear Grenz zone and blast-like atypical cells in the dermis, findings more consistent with BPDCN.

Next, Merkel cell carcinoma (MCC) was also included in our differential diagnosis due to its presentation as reddish nodules. However, dermoscopy of MCC often reveals polymorphous vessels or milky-red areas, which were not observed in our case [12]. Histologically, while MCC and BPDCN share some overlapping features, the positive CD123 expression in our case helped exclude MCC [13].

Finally, we considered basal cell carcinoma (BCC), a common skin malignancy that can present with shiny nodules. On dermoscopy, BCC typically shows arborizing vessels and blue-gray ovoid nests or globules, none of which were observed in this case [14]. Histologically, BCC is characterized by peripheral palisading of tumor cells and retraction artifacts, which were absent. The presence of blast-like atypical cells and positivity for CD123 led us to conclude that BCC was not a likely diagnosis.

By integrating both dermoscopic and histological evidence, we were able to confidently exclude these differential diagnoses and confirm the diagnosis of BPDCN. This combination of diagnostic tools highlights the value of a comprehensive approach in cases of rare and aggressive tumors such as BPDCN.

## Conclusions

This case highlights the significance of early recognition and diagnosis of BPDCN, particularly through dermoscopic examination. The identification of densely clustered reddish-purple dots on dermoscopy, corresponding to histological hemorrhages and small vessel proliferation, emphasizes the potential utility of this non-invasive technique in aiding the diagnosis of BPDCN. Given the aggressive nature and poor prognosis of BPDCN, especially in elderly patients, early diagnosis is essential for improving clinical outcomes. This case underscores the importance of integrating advanced diagnostic tools, such as dermoscopy, in the evaluation of suspicious skin lesions, potentially leading to earlier intervention and better patient management.
